# GFSeeker: a splicing-graph-based approach for accurate gene fusion detection from long-read RNA sequencing data

**DOI:** 10.1093/bib/bbaf702

**Published:** 2026-01-07

**Authors:** Bingyan Wang, Heng Hu, Runtian Gao, Guohua Wang, Tao Jiang

**Affiliations:** College of Computer and Control Engineering, Northeast Forestry University, Harbin 150040, China; College of Life Sciences, Northeast Forestry University, Harbin 150040, China; College of Life Sciences, Northeast Forestry University, Harbin 150040, China; College of Computer and Control Engineering, Northeast Forestry University, Harbin 150040, China; College of Life Sciences, Northeast Forestry University, Harbin 150040, China; School of Computer Science and Technology, Harbin Institute of Technology, Harbin 150001, China; School of Computer Science and Technology, Harbin Institute of Technology, Harbin 150001, China

**Keywords:** gene fusion, long-read RNA sequencing, splicing-graph, dual re-alignment validation

## Abstract

Gene fusions are critical oncogenic drivers and therapeutic targets in diverse cancers. Long-read ribonucleic acid sequencing (RNA-seq) offers an unprecedented opportunity to resolve the full-length structure of fusion isoforms, but its high intrinsic error rates pose significant challenges to the precise identification of true fusion events. Here, we developed GFSeeker, an innovative splicing-graph-based computational framework for accurate gene fusion detection from long-read RNA-seq. GFSeeker employs a unique pipeline based on a splicing graph reference and a dual re-alignment validation to effectively overcome data noise from high error rates. Benchmarking across simulated, non-tumor, and cancer cell line datasets demonstrated GFSeeker’s state-of-the-art performance, achieving 6%–15% higher F1 score compared to existing methods. Notably, GFSeeker successfully identified the known fusion event, *MATN2–POP1*, in the MCF-7 cancer cell line, missed by other tools, highlighting its superior sensitivity in resolving complex fusion events. These results validate GFSeeker as a powerful and reliable tool for gene fusion discovery, heralding its significant potential to advance cancer research and precision diagnostics.

## Introduction

Gene fusions, which result from genomic structural rearrangements or aberrant transcriptional processes, are key molecular events and oncogenic drivers in diverse cancers [1–3]. These alterations can produce functionally abnormal chimeric proteins or place key oncogenes under the control of potent ectopic regulatory elements, thereby profoundly altering cellular signaling pathways [2, 4, 5]. Iconic cases, such as *BCR–ABL1* found in chronic myeloid leukemia (CML) [6, 7] and *TMPRSS2–ERG* in prostate cancer [8, 9], have become effective therapeutic targets and critical diagnostic and prognostic markers [10, 11]. Consequently, the precise identification of gene fusions has become a critical task in genomics-guided precision oncology and continues to drive the development of novel targeted therapies [12].

Transcriptome sequencing, particularly ribonucleic acid sequencing (RNA-seq), has emerged as the preferred technology for detecting these fusion events [13]. Over the past decade, computational tools based on short-read RNA-seq have been the dominant strategy, primarily divided into alignment-based (e.g. STAR-Fusion [14]) and assembly-based (e.g. JAFFA [15]) methods. However, the inherent limitations of short-read sequencing (typically <300 bp) restrict their ability to resolve complex fusion transcripts. Both strategies rely on short reads that span the fusion junction, but they are often hindered by alignment ambiguity when dealing with repetitive regions or genes with complex splicing patterns, making it difficult to reconstruct the full structure of fusion isoforms [16, 17].

In recent years, the rise of long-read sequencing (LRS) technologies, represented by Pacific Biosciences (PacBio) [18] and Oxford Nanopore Technologies (ONT) [19], has provided a powerful alternative for gene fusion detection. By generating reads that can span entire transcripts, this technology offers an unprecedented opportunity to capture the full-length structure of fusion isoforms, promising to overcome the bottlenecks of short-read methods [20, 21]. Furthermore, innovative strategies such as MAS-Iso-seq [22] and R2C2 [23] have been developed to improve the accuracy of LRS, providing a more robust foundation for precise transcript analysis. Despite their significant advantages, LRS data also introduce unique analytical challenges [24], including high intrinsic error rates (typically 5%–15%) and the difficulty of distinguishing true biological fusions from technical artifacts. While a series of tools have been developed to detect gene fusions from LRS data [25–29], accurately and efficiently characterizing the full details of fusion events, particularly achieving precise breakpoint localization and complete isoform reconstruction, remains an active area of research.

To address these challenges, we propose applying a splicing-graph model to this problem. A splicing-graph can compactly represent all transcripts possible of a gene [30]. Given that graph-based models have been widely applied in fields such as variant detection [31, 32] and pangenomics [33–35], and that aligning sequencing reads to a splicing graph can effectively identify alternative splicing events [36]. We believe applying this model to long-read RNA-seq data is a novel and highly promising direction for gene fusion detection.

Here we present GFSeeker, an innovative computational framework designed for the precise detection and deep characterization of gene fusions from long-read RNA-seq data. GFSeeker uses a splicing-graph-based methodology and a rigorous multi-stage validation pipeline to capture complex fusion structures with high resolution, thereby effectively distinguishing true signals from technical noise. Our benchmarking results show that GFSeeker achieves leading performance across diverse datasets and successfully identifies biologically validated fusions missed by other methods, demonstrating its great potential to play an important role in future cancer research and clinical diagnostics.

## Materials and methods

### The GFSeeker workflow

**Figure 1:**
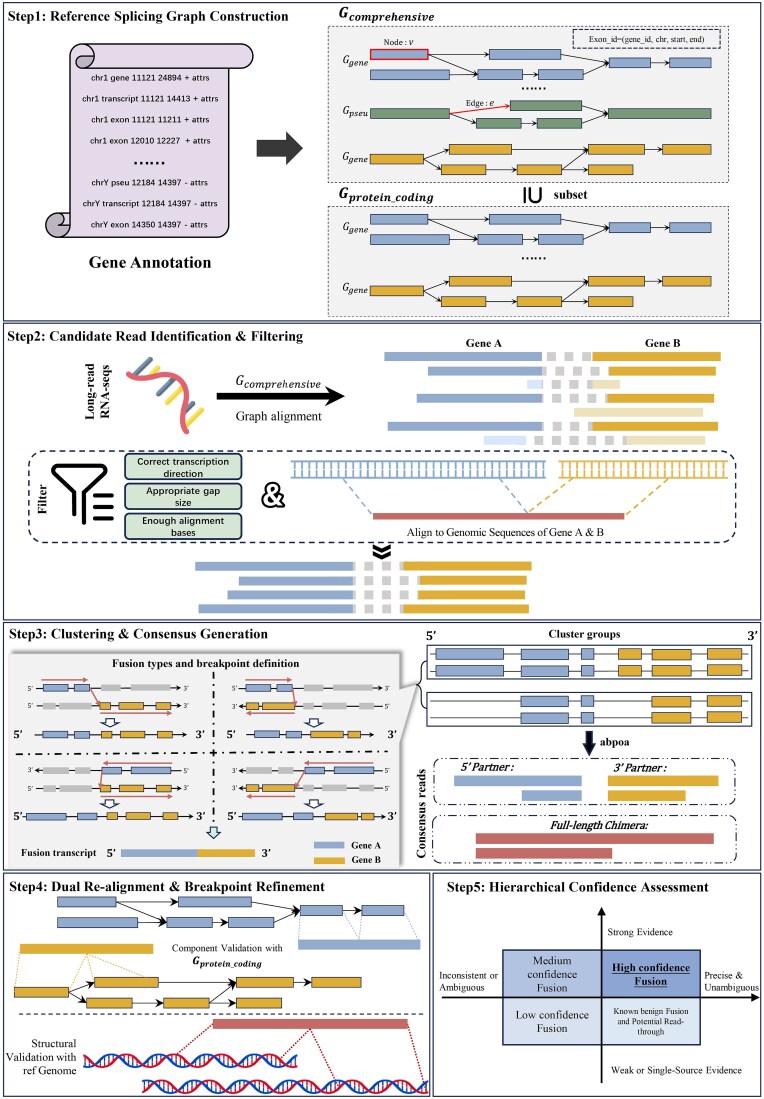
The overall workflow of GFSeeker. GFSeeker follows a five-step process for gene fusion detection. Step 1: reference splicing graph construction. Based on gene annotations, a CRSG (Gcomprehensive) containing all transcripts and a PRSG (Gprotein_coding) restricted to protein-coding genes are constructed. Step 2: candidate read identification and filtering. Candidate reads spanning multiple genes are identified from graph alignment. These are then filtered for consistency and validated by re-aligning to a dynamically built custom reference to precisely confirm the fusion junction. Step 3: clustering and consensus generation. Candidate reads are clustered based on their breakpoints and alignment paths. Three types of high-fidelity consensus sequences are generated for each cluster. Contains an introduction to the four types of fusion and breakpoints. Step 4: dual re-alignment and breakpoint refinement. The validity of fusion events is cross-validated by re-aligning the consensus sequences against both the PRSG and the linear reference genome, enabling precise refinement of breakpoint locations. Step 5: Hierarchical confidence assessment. All validation evidence is integrated to classify candidate fusions into different confidence tiers based on evidence strength, consistency, and comparison against known artifacts.

The GFSeeker framework identifies gene fusions through a five-step process, as illustrated in Fig. 1. The workflow begins by constructing two reference splicing graphs from gene annotations: a comprehensive reference splicing graph (CRSG) and a protein-coding reference splicing graph (PRSG). Long reads are then aligned to the CRSG to identify candidate fusion-supporting reads, which are subsequently clustered and refined through a multi-stage validation pipeline, including dual re-alignment and a hierarchical confidence assessment. This design aims to deeply leverage the full-length transcript information provided by LRS, thereby enabling the simultaneous identification of both known and novel gene fusion events from complex transcriptomic backgrounds with high sensitivity and precision.

### Reference splicing graph construction and formal definition

To accurately identify gene fusion events, we first constructed a reference transcriptome based on a splicing graph. Formally, a splicing graph is defined as a directed acyclic graph (DAG), denoted as $G=\left(V,E\right)$, where $V$ represents the set of exon nodes and $E$ represents the set of splicing edges, as visually defined in Fig. 1, Step 1. This graph structure compactly represents the complex splicing patterns of all known transcripts within a gene.

We construct the graphs using authoritative public databases, GENCODE gene annotations in GFF3 format. We primarily focused on protein-coding genes to mitigate the analytical challenges arising from the complex and sporadic splicing events that are prevalent in non-coding RNAs and pseudogenes. Each exon is abstracted as a unique node $v\in V$, formally defined by a four-tuple to capture its genomic location and gene identity:


(1) \begin{equation*} v=\left( gen{e}_{id}, chr, start, end\right) \end{equation*}

where ${gene}_{id}$ is the unique gene identifier provided by the database, $chr$ is the chromosome on which it resides, and $start$ and $end$ are its precise start and end coordinates on the genome, respectively. This refined node definition ensures that even different exons with overlapping genomic coordinates (e.g. ‘cassette exons’ resulting from alternative splicing) are distinguished as separate nodes in the graph, as long as their boundaries or parent transcripts differ.

A directed edge $e=\left({v}_i,{v}_j\right)$ exists if exon ${v}_i$ is immediately followed by exon ${v}_j$ in a known transcript isoform. Notably, to simplify downstream alignment and analysis processes, all exon node sequences and connections reconstructed from graph paths are normalized to the positive strand direction. This greatly facilitates the subsequent identification and comparison of gene fusion breakpoints.

Based on the above definitions, we first construct an independent Gene Subgraph ${G}_g=\left({V}_g,{E}_g\right)$ for each gene $g$. Here, ${V}_g\in V$ is the set of all exon nodes contained within gene $g$, and ${E}_g\in E$ is the set of splicing edges connecting these internal nodes. By integrating these independent gene subgraphs and adhering to the graphical fragment assembly (GFA) specification (https://gfa-spec.github.io/GFA-spec/GFA1.html), we generate two core reference graphs:

(1) The CRSG, formally denoted as ${G}_{comprehensive}$, is constructed to represent the full diversity of splicing structures within a given reference. This graph is ‘comprehensive’ in relation to a standard gene annotation, thereby encapsulating the complete topological structure of the entire annotated transcriptome. Using this comprehensive graph for the initial alignment maximizes the sensitivity of detection, ensuring that potential true fusions involving non-coding genes or pseudogenes are not prematurely discarded. Let $A$ be the set of all annotated genes, the construction of ${G}_{comprehensive}=\left({V}_{comprehensive},{E}_{comprehensive}\right)$ can be formally expressed as the union of the vertex sets and edge sets from all gene subgraphs:

(2) \begin{equation*} {\displaystyle \begin{array}{c}{V}_{comprehensive}=\bigcup_{g\in A}{V}_g\end{array}} \end{equation*}

(3) \begin{equation*} {\displaystyle \begin{array}{c}{E}_{comprehensive}=\bigcup_{g\in A}{E}_g\end{array}} \end{equation*}

(2) To subsequently improve the precision of the validation step and focus on events with higher potential for functional impact, we constructed the PRSG. The PRSG, formally denoted as ${G}_{protein\_ coding}$, is a curated subset of the CRSG, containing only the subgraphs related to protein-coding genes. It provides a more focused reference with a higher signal-to-noise ratio for subsequent analyses and serves as a key resource for the secondary, higher-precision validation of candidate gene fusion events identified in the initial screening. Let $P$ be the set of all protein-coding genes ($P\subseteq A$). ${G}_{protein\_ coding}=\left({V}_{protein\_ coding},{E}_{protein\_ coding}\right)$, is defined as:

(4) \begin{equation*} {\displaystyle \begin{array}{c}{V}_{protein\_ coding}=\bigcup_{g\in P}{V}_g\end{array}} \end{equation*}

(5) \begin{equation*} {\displaystyle \begin{array}{c}{E}_{protein\_ coding}=\bigcup_{g\in P}{E}_g\end{array}} \end{equation*}

Furthermore, to enable rapid retrieval of a specific gene from an aligned node, we use a hash map-based index to efficiently retrieve gene information from any alignment position, which effectively improves the speed and accuracy of gene identification during the alignment process.

### Identification and validation of candidate fusion events

The core workflow of this study employs a multi-stage strategy to identify and refine reads supporting gene fusions.

1. **Initial candidate discovery via graph alignment**

The first step of the workflow is to align the raw sequencing data against the previously constructed CRSG (${G}_{comprehensive}$). We employ Minigraph [37], an alignment tool specifically designed for graph structures, to perform this task. This step produces a series of alignments in the graphical alignment format (GAF), which detail the alignment path of each read, its alignment orientation, CIGAR string, and other key information, providing the foundation for identifying inter-gene connections.

1. **Targeted junction validation and filtering**

After the initial graph alignment, GFSeeker first identifies a preliminary pool of candidate reads by parsing the GAF files for alignment paths that span two or more different gene subgraphs. To validate these candidates, GFSeeker performs a comprehensive Targeted Junction Validation. A custom reference sequence consisting of the full genomic sequence of both partner genes is dynamically constructed for all candidate gene pairs and compiled into a single, consolidated reference file to enable efficient validation. Finally, all candidate reads are mapped in a single pass against this precise, consolidated custom reference file using Minimap2 [38] in splice mode.

This validation process is designed to minimize false positives caused by sequencing errors, sequence homology, or alignment ambiguities. It confirms the validity of a candidate by ensuring it simultaneously satisfies the following stringent criteria, including: structural consistency constraints, transcriptional direction consistency, minimum block length, validation of the fusion junction, and removal of alignment anomalies. The detailed description for each criterion is provided in the supplementary note.

### Clustering of fusion candidates and consensus transcript generation

To precisely characterize distinct fusion isoforms, validated reads undergo a two-stage clustering process that involves clustering the candidate reads and generating an unbiased consensus sequence for each cluster.

First, we ensure that only reads supporting the exact same fusion event are grouped together. The first phase is Breakpoint-based Coarse Clustering, we determine an approximate breakpoint region for each candidate read. This region is defined by the two boundary exons that constitute the fusion junction (i.e., the last exon of the 5′ partner gene and the first exon of the 3′ partner gene). The principle of this breakpoint definition is visually represented in the left panel of Fig. 1, Step 3, where the junction connecting the two gene partners indicates the location of the fusion breakpoint. Specifically, GFSeeker determines whether the breakpoint is before or after a specific exon using the following rule: for each fusion candidate gene-pair A–B, if gene A is aligned in a forward path or gene B in a reverse path, the breakpoint is located at the maximum position of the exons in the path; if gene A is aligned in a reverse path or gene B in a forward path, the breakpoint is at the minimum position. We then perform rapid initial clustering based on the genomic coordinates of these breakpoint regions using Manhattan distance to group reads with nearby breakpoints. The Manhattan distance is calculated as:


(6) \begin{equation*} {\displaystyle \begin{array}{c} Manhattan=\left|{x}_i-{x}_j\right|+\left|{y}_i-{y}_j\right|\end{array}} \end{equation*}

where $\left({x}_i,{y}_i\right)$ and $\left({x}_j,{y}_j\right)$ are the coordinates of two different fusion breakpoint pairs.

The second clustering phase is path-based fine-grained Clustering. Within each coarse cluster, we perform a more refined secondary clustering. This step is based on the exact path each read traverses in the reference splicing graph. Two reads are assigned to the same final cluster only if their sequence of traversed nodes (exons) is identical. Through this strategy, each resulting cluster is defined by a unique ‘breakpoint-path’ identifier, representing a highly specific fusion isoform.

To eliminate the impact of individual sequencing errors and construct a high-fidelity representative sequence for the fusion isoform, we utilize the abPOA tool [39], which performs Partial Order Alignment. For each ‘breakpoint-path’ cluster, we generate three key types of consensus sequences for subsequent detailed validation: 5′ partner fragment sequence; 3′ partner fragment sequence; Full-length chimeric transcript sequence.

### Breakpoint refinement and confidence assessment via re-alignment

Although splicing-graph-based alignment can detect fusion candidates with high sensitivity, the resulting breakpoint locations are constrained by the graph’s topology and may deviate from the true genomic splice sites. To achieve base-level precision and assess reliability, we designed a comprehensive validation workflow.

**Step 1: Dual re-alignment validation**

The consensus sequence of each candidate fusion event must undergo the following two parallel validation processes, to cross-validate the fusion structure:

(1) **Component validation on the PRSG:** We re-align the 5′ and 3′ partner consensus sequences separately to the PRSG (${G}_{protein\_ coding}$). This step serves a dual purpose: first, it rigorously re-confirms the identity of the fusion partners, particularly verifying if they are protein-coding genes, which is crucial for assessing the potential pathogenicity of the fusion event. Second, it ensures that the error-corrected consensus sequences still perfectly match known, valid transcript exon paths, thereby increasing the credibility of the fusion structure.
(2) **Structural validation on the linear reference genome:** We align the full-length consensus fusion transcript sequence to a standard human reference genome (e.g. hg38) using Minimap2. This linear, annotation-independent alignment can accurately locate the splice junction spanning the two genes and verify if the connection conforms to canonical splice signals (e.g. GT-AG), providing the most direct genomic structural evidence for the fusion event.

**Step 2: Breakpoint refinement**

After completing the re-alignments, we refine the breakpoint positions. Because the inherent error rate of LRS can lead to imprecise alignment ends, if an inferred breakpoint is located near a known splice site (e.g. within 30 bp), we correct the breakpoint to this exon boundary. This approach and the 30 bp threshold are consistent with practices in other state-of-the-art tools that use a defined search window to handle positional uncertainties and refine breakpoints near annotated splice junctions [25, 29]. This step aims to rectify deviations caused by sequencing or alignment errors to achieve base-level precision, as true structural rearrangements, while occurring in intronic regions, often preserve splice sites, manifesting at the transcript level as the joining of exon ends.

**Step 3: Hierarchical confidence assessment**

Finally, GFSeeker integrates all evidence to classify fusions. This classification process includes a comparison against the CTAT Human Fusion Lib database (https://github.com/FusionAnnotator/CTAT_HumanFusionLib/wiki) to identify known red herrings (e.g. fusions commonly found in normal tissues, known read-through events, etc.). All candidate events are then classified into the following tiers:

(1) **High confidence:** events are classified as high confidence if they meet one of two criteria: (i) they are dually validated against both the PRSG and the linear genome with consistent breakpoints (<30 bp apart), or (ii) they involve unambiguously separated genes (distant >100 kbp) or separated by at least one other protein-coding gene, a structure that rules out read-through artifacts.
(2) **Medium confidence:** events with strong dual-validation evidence, but there is a certain deviation in their breakpoint positions (exceeding the preset threshold). This often indicates a potential true fusion event but with ambiguity or potential non-canonical splicing at the precise junction.
(3) **Low confidence:** events supported by strong evidence from only a single validation dimension. This includes fusions that validate only against the linear genome (potentially involving non-coding regions) or only against the PRSG (common for inter-chromosomal or structurally complex events).
(4) **Potential artifacts/read-through:** this includes events flagged as known artifacts by the database, as well as suspected read-through transcripts. The latter are characterized by fusions between adjacent, co-oriented genes on the same chromosome with no intervening protein-coding genes.

This system validates each candidate’s structure, distinguishing high-confidence, potentially pathogenic novel events from those that are known or suspected to be benign events to provide an accurate and interpretable list. Details of the metrics for the evaluation and experimental implementation are provided in the supplementary notes.

## Results

### Performance evaluation on simulated datasets

To evaluate GFSeeker’s performance, we used pbsim3 [40] to construct simulated datasets reflecting both PacBio and ONT error models across various sequencing depths (10×, 30×, 50×) and error rates (5%, 10%, 15%). The simulation included a positive control with 146 known tumor fusion events and a negative control designed to mimic a non-tumor transcriptome. We benchmarked GFSeeker against five leading long-read fusion detection tools: JAFFAL, GFHunter, LongGF, CTAT-LR-Fusion, and FusionSeeker. Detailed construction methods and benchmark results are provided in the Supplementary Table S1 and supplementary notes.

1. **GFSeeker improved gene-pair level detection performance**

**Figure 2:**
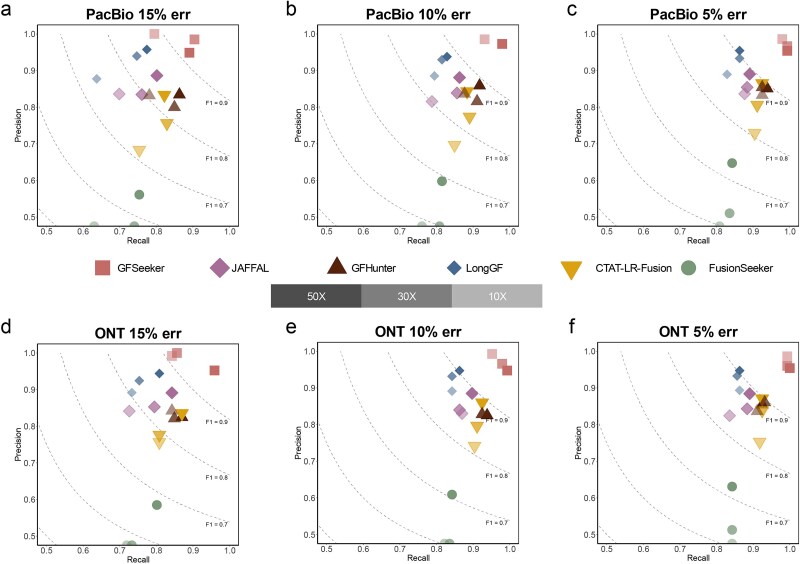
Comparison of the gene-pair level fusion detection performance of GFSeeker with five other tools on simulated datasets. The precision-recall plots for six tools are shown for simulated (a–c) PacBio and (d–f) ONT data with 15%, 10%, and 5% error rates, respectively. Points located on the *x*-axis indicate precision values below 0.5.

As shown in Fig. 2 and Supplementary Table S2, GFSeeker demonstrated superior gene-pair level detection performance compared to the other five tools across all 18 simulated conditions, achieving an average F1 score exceeding 95% on PacBio data and 96% on ONT data. Its strength was particularly evident under the most challenging conditions (10× coverage, 15% error rate). In this scenario, while tools like GFHunter and CTAT-LR-Fusion introduced 23 and 38 false positives respectively, GFSeeker consistently reported eight or fewer while maintaining near-perfect precision (99%–100%). This exceptional ability to distinguish signal from noise, attributed to its stringent multi-stage validation pipeline, resulted in a significantly higher F1 score. In stark contrast, FusionSeeker’s performance was hindered by a large number of false positives, which we hypothesize may stem from its permissive use of intronic alignments as fusion evidence.

1. **GFSeeker infers more precise gene fusion event breakpoints**

**Figure 3:**
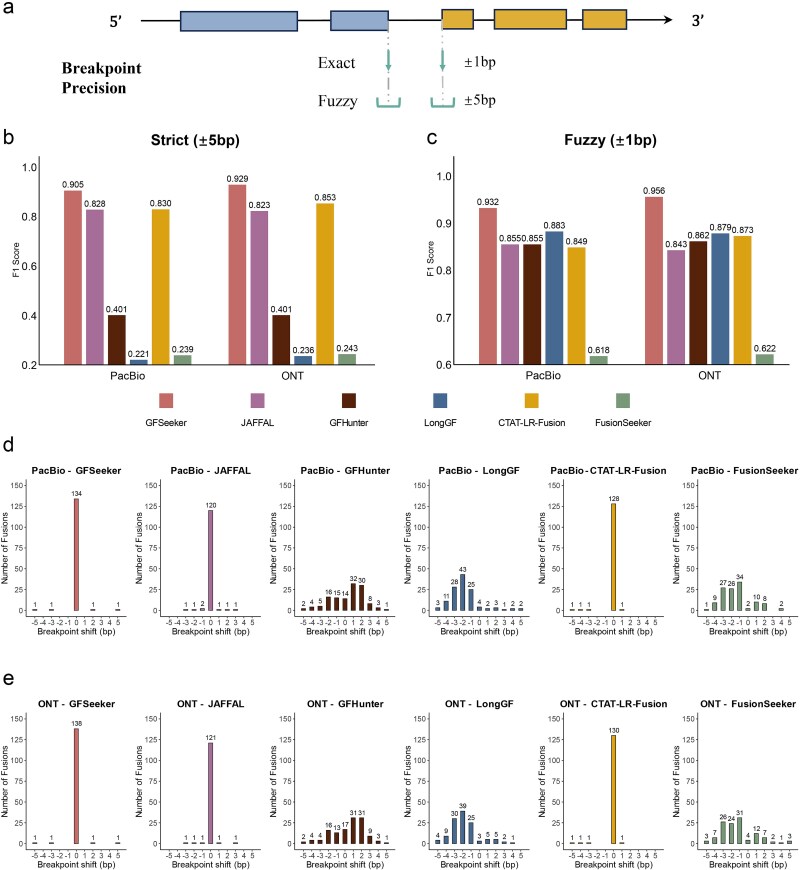
Breakpoint location accuracy analysis. Panel (a) schematically explains the evaluation modes for breakpoint precision. Panels (b) and (c) show the breakpoint prediction F1 scores for each tool under strict and fuzzy modes, respectively, on data with 30× coverage and a 15% error rate. Panels (d) and (e) then present the detailed breakpoint shift distributions on PacBio and ONT sequencing data.

In addition to gene-pair detection, GFSeeker also excelled in breakpoint localization accuracy. As shown in Fig. 3b and c and Supplementary Table S3, GFSeeker achieved the highest F1 score among all tested tools in both ‘Strict’ (±1 bp) and ‘Fuzzy’ (±5 bp) evaluation modes (Fig. 3a).

To further investigate this high precision, we analyzed the breakpoint shift distribution (Fig. 3d and e). This analysis revealed that a significant majority of GFSeeker’s predicted breakpoints had a shift of 0 bp (>97% on both PacBio and ONT data), indicating perfect base-level accuracy. In contrast, other tools like GFHunter and FusionSeeker exhibited a much wider distribution of shifts. This exceptional precision is primarily attributable to GFSeeker’s re-alignment-based breakpoint refinement strategy.

### Benchmark on real non-tumor datasets

To evaluate the detection performance of each tool on real data, we used three non-tumor datasets from the human sample HG002, generated using PacBio Iso-Seq, ONT direct RNA (dRNA), and ONT cDNA platforms. Detailed statistics of datasets are provided in Supplementary Table S8. Gene fusion is an exceedingly rare event in non-tumor tissues; therefore, to assess specificity, any fusion event reported by a tool in this context is considered a false positive. This allows for a direct comparison of each tool’s ability to suppress artifacts.

As shown in Table 1, all tools reported a significantly higher number of candidate fusion events on the cDNA and Iso-seq datasets compared to the dRNA dataset. This phenomenon did not show a clear positive correlation with sequencing throughput. By examining the detections from each tool, we found that the enriched candidate events in the cDNA and Iso-seq data mostly occurred between genes that were co-located on the same chromosome and in close proximity. We speculate that these false positives originate from technical artifacts generated during the library construction process common to both technologies. Specifically, since library preparation for both cDNA and Iso-seq involves reverse transcription and PCR amplification, template switching can occur due to incomplete extension or breakage of the template strand, which then incorrectly uses another RNA molecule as a template for continued synthesis. This process generates chimeric cDNA molecules, which are highly prone to being misidentified as true gene fusions in subsequent analyses.

**Figure 4:**
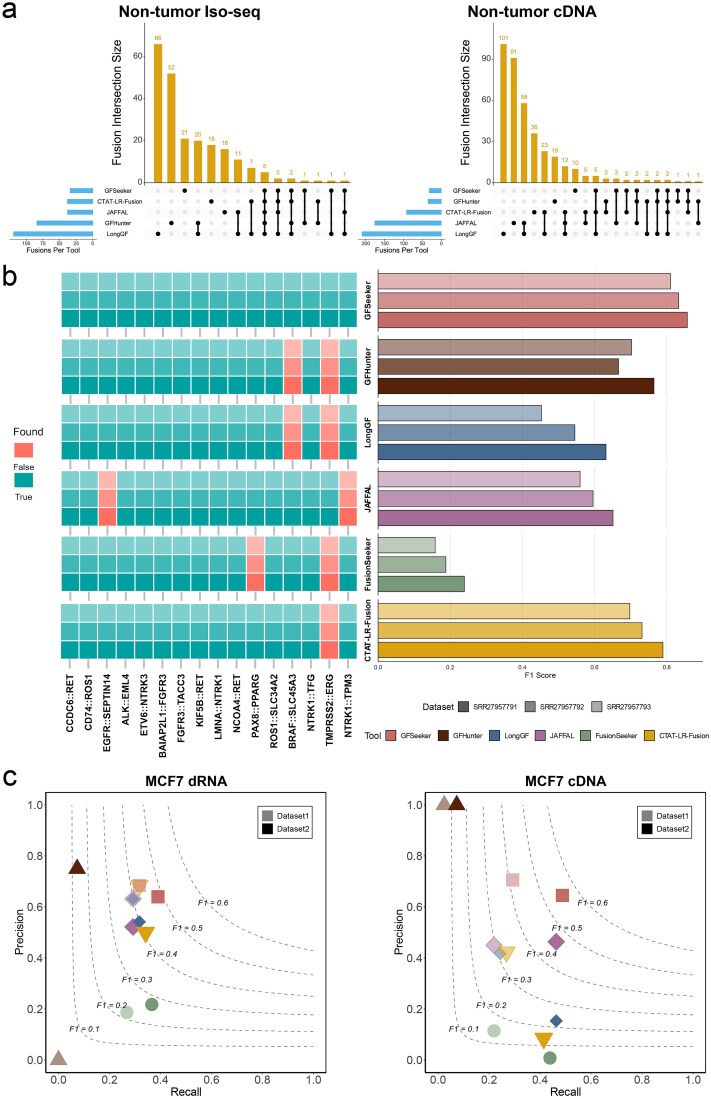
Performance evaluation on real non-tumor and cancer datasets. (a) Intersection of tool detections in non-tumor samples. The upset plot shows the intersection of candidate fusion events reported by the tools (excluding FusionSeeker) on the HG002 Iso-Seq and cDNA datasets. (b) Performance on PacBio Iso-Seq datasets. The heatmap shows the detection status of 16 gold-standard fusion events in the Seraseq reference sample across three technical replicates. The adjacent bar plot shows the corresponding mean F1 scores. (c) Precision-recall graphs of all tools for gene fusion detection on MCF-7 dRNA and cDNA datasets.

In this test, GFSeeker demonstrated outstanding specificity, reporting the lowest total number of candidate events across all three datasets. In contrast, some tools generated a large number of false positives under lenient filtering criteria. For example, FusionSeeker reported up to 1355 candidate events on the Iso-seq dataset, an order of magnitude higher than other tools, suggesting its results may contain substantial background noise. To ensure the validity of the comparative analysis, we excluded FusionSeeker and analyzed the intersection of the detection results from the remaining tools on the Iso-seq and cDNA datasets (Fig. 4a). The results showed that the vast majority of candidate events were reported by only a single tool, with events supported by multiple tools being extremely rare. The number of events dropped sharply when requiring detection by two or more tools, and only two events were unanimously identified by all five tools. This stark disparity confirms our core assertion: in non-tumor samples, true fusion events are exceedingly rare, and most detected signals arise from differences in how various algorithms tolerate transcriptomic complexity and sequencing errors.

Particularly, GFSeeker’s stringent filtering did not compromise its sensitivity to real biological events. It successfully identified two literature-validated transcriptional events known to exist in normal tissues: the *CTBS–GNG5* fusion [41] (detected in all three datasets) and the *SEC31B–NDUFB8* read-through event [42]. GFSeeker correctly classified these as ‘Potential Artifacts/Read-through’. Among the other benchmarked tools, most failed to detect these events at all. Only GFHunter reported the *CTBS–GNG5* event but labeled it less specifically as a ‘Potential Fusion’. This highlights GFSeeker’s unique ability to not only filter out technical noise but also accurately capture and classify known biological complexities, reflecting its algorithm’s superior comprehension of RNA complexity.

### Performance validation on real cancer datasets

To evaluate the tools’ detection capabilities in a known context, we used a commercial reference RNA sample (Seraseq Fusion RNA Mix v4) containing 16 known fusion transcripts that was sequenced in three technical replicates using the MAS-Iso-seq method. Detailed statistics of datasets are provided in Supplementary Table S8. In these datasets, GFSeeker demonstrated a perfect recall rate (Supplementary Table S4), successfully detecting all 16 reference fusion events (left heatmap of Fig. 4b) and achieving the highest overall F1 score among all tested tools (91.4%, 88.9%, and 86.5%, respectively). In contrast, other tools had several missed detections. In addition to its superior performance in gene-pair detection, GFSeeker also led in breakpoint localization accuracy (Supplementary Table S5). As shown in the right bar chart of Fig. 4b, when evaluated using the Fuzzy mode, its F1 score for breakpoint prediction was the highest among all tools, indicating its ability to precisely locate fusion junction sites. Notably, the *TMPRSS2–ERG* fusion, an event that is often difficult to detect, was stably identified only by GFSeeker and JAFFAL.

To further validate performance in a real-world scenario with an unknown background, we used sequencing data from the well-studied MCF-7 human breast cancer cell line [43]. We constructed a benchmark ground truth set of 41 high-confidence fusion events by curating published studies [44–49] and evaluated all tools on several SG-NEx ONT datasets [50]. Detailed statistics of datasets are provided in Supplementary Table S8. These datasets included two different cDNA library types and two dRNA biological replicates to comprehensively evaluate algorithmic robustness. The benchmark results for each dataset and full ground truth list can be found in Supplementary Tables S6 and S7.

**Table 1:** Reported fusions on HG002 non-tumor datasets.

HG002	GFSeeker	LongGF	JAFFAL	CTAT-LR-Fusion	FusionSeeker	GFHunter
cDNA^a^	**32**	210	176	92	1614	35
dRNA^a^	**0**	4	5	1	21	1
Iso-seq^b^	**21**	119	38	38	1355	84

^a^The minimum number of supported fusion events is 3.

^b^The minimum number of supported fusion events is 6. Bold values indicate the lowest number of reported fusions in each dataset.

**Figure 5:**
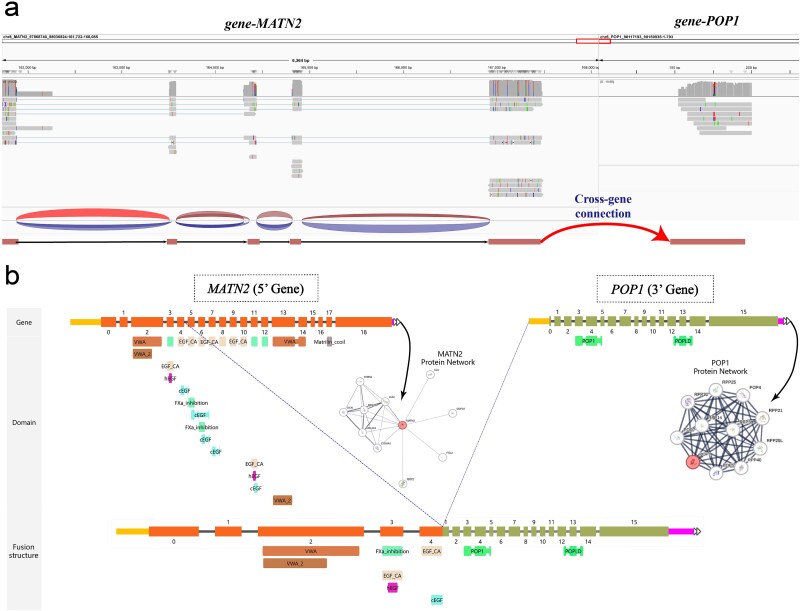
Validation and structural analysis of the MATN2-POP1 fusion event exclusively detected by GFSeeker in the MCF-7 cell line. (a) An IGV (integrative genomics viewer) visualization of long-read alignments supporting the MATN2-POP1 fusion event. (b) A schematic diagram of the MATN2-POP1 fusion event, adapted from the ChimerDB database [51]. The diagram shows the original exon and protein domain composition of MATN2 and POP1, the resulting fusion structure, and the respective protein interaction networks for each protein.

Across all MCF-7 datasets, GFSeeker demonstrated superior and robust performance, consistently achieving a leading F1 score (Fig. 4c). Whether on cDNA data, which is prone to chimeric artifacts, or on dRNA data with its unique error profile, GFSeeker showed strong adaptability and excellent false-positive control while consistently detecting the highest number of true positives. In contrast, the performance of other tools fluctuated more significantly; for example, GFHunter’s recall was notably low across all datasets, while other tools were generally accompanied by more false positives. Crucially, GFSeeker was the only tool to identify the literature-validated *MATN2–POP1* fusion event in the MCF-7 datasets (Fig. 5a) [46], which was supported by 12 and 7 long reads in the cDNA and dRNA datasets, respectively. Through manual inspection and re-alignment analysis, we confirmed that reads supporting this event showed a canonical splicing pattern highly consistent with the structure recorded in public databases (Fig. 5b) [51]. This exclusive detection highlights the exceptional sensitivity of GFSeeker’s splicing-graph-based model in resolving complex or low-abundance fusion events that other methods miss.

## Discussion

This study introduces GFSeeker, an innovative computational framework designed for the precise detection and in-depth characterization of gene fusions from long-read RNA-seq data. Through its innovative splicing-graph-based methodology and rigorous multi-stage validation strategy, GFSeeker achieves state-of-the-art accuracy and reliability. Extensive benchmarking on simulated and real-world cancer datasets demonstrates its ability to identify true fusion events with high sensitivity while maintaining an extremely low false-positive rate. GFSeeker’s outstanding performance is attributed to follow three core designs: (i) GFSeeker’s splicing-graph-based model exhibits exceptional sensitivity, uncovering fusion events in complex cancer transcriptomes that prove elusive to other detection methods. (ii) Its breakpoint refinement strategy, which anchors inferred breakpoints to known splice sites, effectively corrects for deviations caused by sequencing or alignment errors. This enables the precise inference of the fusion junction, achieving zero-deviation, single-base precision for over 97% of its predictions in simulated data. (iii) Its unique dual re-alignment validation pipeline (validating against both the PRSG and the linear reference genome) is key to its extremely low false-positive rate. This was fully demonstrated in the analysis of non-tumor datasets, where GFSeeker reported far fewer false positives than other tools.

A deeper analysis of GFSeeker’s performance further highlights its unique advantage in comprehending transcriptome complexity. In non-tumor samples, a core challenge is not only filtering technical noise but also distinguishing it from real, low-abundance biological events like read-through transcription. GFSeeker accomplished the former with the lowest false-positive rate, and it also successfully identified and accurately annotated literature-validated transcriptional events known to exist in normal tissues, such as *CTBS–GNG5* and *SEC31B–NDUFB8*, which were missed or misclassified by most other tools. This demonstrates that GFSeeker can provide a more refined and biologically accurate view of the transcriptome than other tools. In the analysis of cancer datasets, GFSeeker’s advantage is evident in its ability to discover events of significant clinical interest. For example, its exclusive detection of the *MATN2–POP1* fusion in the MCF-7 cell line not only re-confirms its high sensitivity but also underscores its potential for clinical application. This fusion event is often missed by other tools, potentially due to low expression levels or a complex breakpoint structure. The resulting *MATN2–POP1* chimeric transcript, as detailed in Fig. 5b, would likely produce an aberrant protein. Such an alteration could disrupt the normal functions of the parent proteins and subsequently perturb their established protein interaction networks, thereby implicating it as a potential cancer-driving event.

Despite its outstanding performance, GFSeeker has certain limitations. First, its performance is highly dependent on the quality of the gene annotations used to build its reference splicing graph. For well-annotated genomes like *Homo sapiens*, it can achieve extremely high accuracy, but its performance may be constrained when analyzing species with incomplete or inaccurate annotations. More effort should be paid to integrating de novo transcript assembly methods to augment the splicing graph, thereby enabling the detection of fusions involving novel or unannotated genes. Second, while GFSeeker effectively reconstructs fusion isoforms, its capabilities have not been exhaustively benchmarked on highly complex multi-gene fusions or events involving extensive, unannotated alternative splicing near the breakpoint. Future research could enhance the graph model and validation logic to better characterize these more intricate transcriptional rearrangements, as well as explore its application in the single-cell field and integrate it with clinical variant annotation pipelines to further bridge the gap between genomic discoveries and therapeutic applications.

In summary, GFSeeker represents a significant advancement in the field of gene fusion detection from long-read RNA-seq data. Its capacity to provide highly accurate and detailed characterizations of gene fusion events heralds its great potential to accelerate discoveries in cancer genomics and to become an essential tool in the era of genomics-guided precision oncology.

**Key Points**

- GFSeeker utilizes a reference splicing graph constructed from gene annotations, replacing the conventional linear genome. This splicing-graph-based approach is more suitable for accurately representation of complex transcript isoforms, significantly improving alignment sensitivity for long reads across complex splicing patterns.
- GFSeeker implements an innovative two-stage clustering strategy that combines breakpoint proximity with graph path identity. This method accurately distinguishes different fusion isoforms and overcome the effects of the various errors, enabling high-fidelity consensus generation for each unique event.
- GFSeeker introduces a unique dual re-alignment pipeline to ensure high reliability. To confirm gene components and structural integrity, consensus sequences are cross-validated against both the PRSG and the linear genome. In addition, GFSeeker supports comparison against known artifact databases and genomic distance to classify candidates, providing users with a clear interpretation of the results.
- GFSeeker demonstrates superior gene fusion detection performance across different long-read platforms and library preparation methods. It maintains exceptional precision and recall even under challenging conditions of low coverage and high error rates, providing a reliable solution for fusion discovery from diverse transcriptomic datasets.

## Supplementary Material

Supplementary_materials_bbaf702

## Data Availability

The links to the reference genome, gene annotation, public datasets used for benchmarking are available in Supplementary Table S9. All the version and download URL of tools used in this study are listed in Supplementary Table S10. The benchmarked commands, simulation workflow, the fusion detection commands of all tools are available in Supplementary Notes. The GFSeeker code is available at https://github.com/EmotionWaste/GFSeeker.

## References

[1] Edwards P . Fusion genes and chromosome translocations in the common epithelial cancers. *J Pathol* 2010;220:244–54. 10.1002/path.2632 19921709

[2] Mertens F, Johansson B, Fioretos T. et al. The emerging complexity of gene fusions in cancer. *Nat Rev Cancer* 2015;15:371–81. 10.1038/nrc3947 25998716

[3] Foltz SM, Gao Q, Yoon CJ. et al. Evolution and structure of clinically relevant gene fusions in multiple myeloma. *Nat Commun* 2020;11:2666. 10.1038/s41467-020-16434-y 32471990 PMC7260243

[4] Taniue K, Akimitsu N. Fusion genes and RNAs in cancer development. *Noncoding RNA* 2021;7:10. 10.3390/ncrna7010010 33557176 PMC7931065

[5] Yang W, Lee K-W, Srivastava RM. et al. Immunogenic neoantigens derived from gene fusions stimulate T cell responses. *Nat Med* 2019;25:767–75. 10.1038/s41591-019-0434-2 31011208 PMC6558662

[6] Saha V, Young BD, Freemont P. Translocations, fusion genes, and acute leukemia. *J Cell Biochem* 1998;72:264–76. 10.1002/(SICI)1097-4644(1998)72:30/31+<264::AID-JCB32>3.3.CO;2-L 29345835

[7] Martínez-Castillo M, Gómez-Romero L, Tovar H. et al. Genetic alterations in the BCR-ABL1 fusion gene related to imatinib resistance in chronic myeloid leukemia. *Leuk Res* 2023;131:107325. 10.1016/j.leukres.2023.107325 37302352

[8] Mosquera JM, Perner S, Demichelis F. et al. Morphological features of TMPRSS2–ERGgene fusion prostate cancer. *J Pathol* 2007;212:91–101. 10.1002/path.2154 17385188

[9] Wang Z, Wang Y, Zhang J. et al. Significance of the TMPRSS2:ERG gene fusion in prostate cancer. *Mol Med Rep* 2017;16:5450–8. 10.3892/mmr.2017.7281 28849022 PMC5647090

[10] Linardic C . PAX3–FOXO1 fusion gene in rhabdomyosarcoma. *Cancer Lett* 2008;270:10–8. 10.1016/j.canlet.2008.03.035 18457914 PMC2575376

[11] Sun C, Chang L, Zhu X. Pathogenesis ofETV6/RUNX1-positive childhood acute lymphoblastic leukemia and mechanisms underlying its relapse. *Oncotarget* 2017;8:35445–59. 10.18632/oncotarget.16367 28418909 PMC5471068

[12] Heyer EE, Deveson IW, Wooi D. et al. Diagnosis of fusion genes using targeted RNA sequencing. *Nat Commun* 2019;10:1388. 10.1038/s41467-019-09374-9 30918253 PMC6437215

[13] Bruno R, Fontanini G. Next generation sequencing for gene fusion analysis in lung cancer: a literature review. *Diagnostics (Basel)* 2020;10:521. 10.3390/diagnostics10080521 32726941 PMC7460167

[14] Haas BJ, Dobin A, Li B. et al. Accuracy assessment of fusion transcript detection via read-mapping and de novo fusion transcript assembly-based methods. *Genome Biol* 2019;20:213–6. 10.1186/s13059-019-1842-9 31639029 PMC6802306

[15] Davidson NM, Majewski IJ, Oshlack A. JAFFA: high sensitivity transcriptome-focused fusion gene detection. *Genome Med* 2015;7:43–12. 10.1186/s13073-015-0167-x 26019724 PMC4445815

[16] Kumar S, Vo AD, Qin F. et al. Comparative assessment of methods for the fusion transcripts detection from RNA-seq data. *Sci Rep* 2016;6:21597. 10.1038/srep21597 26862001 PMC4748267

[17] Liu S, Tsai W-H, Ding Y. et al. Comprehensive evaluation of fusion transcript detection algorithms and a meta-caller to combine top performing methods in paired-end RNA-seq data. *Nucleic Acids Res* 2016;44:e47–7. 10.1093/nar/gkv1234 26582927 PMC4797269

[18] Roberts RJ, Carneiro MO, Schatz M. The advantages of SMRT sequencing. *Genome Biol* 2013;14:1–4. 10.1186/gb-2013-14-7-405 PMC3953343 23822731

[19] Mikheyev AS, Tin M. A first look at the Oxford nanopore MinION sequencer. *Mol Ecol Resour* 2014;14:1097–102. 10.1111/1755-0998.12324 25187008

[20] Xia Y, Jin Z, Zhang C. et al. TAGET: a toolkit for analyzing full-length transcripts from long-read sequencing. *Brief Bioinform* 2023;14:5935. 10.1038/s41467-023-41649-0 PMC10518008 37741817

[21] Pardo-Palacios FJ, Wang D, Reese F. et al. Systematic assessment of long-read RNA-seq methods for transcript identification and quantification. Nature methods 2024;21:1349–63. 10.1038/s41592-024-02298-3 PMC11543605 38849569

[22] Al’Khafaji AM, Smith JT, Garimella KV. et al. High-throughput RNA isoform sequencing using programmed cDNA concatenation. *Nat Biotechnol* 2024;42:582–6. 10.1038/s41587-023-01815-7 37291427 PMC12236355

[23] Volden R, Palmer T, Byrne A. et al. Improving nanopore read accuracy with the R2C2 method enables the sequencing of highly multiplexed full-length single-cell cDNA. *Proc Natl Acad Sci U S A* 2018;115:9726–31. 10.1073/pnas.1806447115 30201725 PMC6166824

[24] Amarasinghe SL, Su S, Dong X. et al. Opportunities and challenges in long-read sequencing data analysis. *Genome Biol* 2020;21:30. 10.1186/s13059-020-1935-5 32033565 PMC7006217

[25] Davidson NM, Chen Y, Sadras T. et al. JAFFAL: detecting fusion genes with long-read transcriptome sequencing. *Genome Biol* 2022;23:10. 10.1186/s13059-021-02588-5 34991664 PMC8739696

[26] Liu Q, Hu Y, Stucky A. et al. LongGF: computational algorithm and software tool for fast and accurate detection of gene fusions by long-read transcriptome sequencing. *BMC Genomics* 2020;21:793–12. 10.1186/s12864-020-07207-4 33372596 PMC7771079

[27] Chen Y, Wang Y, Chen W. et al. Gene fusion detection and characterization in long-read cancer transcriptome sequencing data with FusionSeeker. *Cancer Res* 2023;83:28–33. 10.1158/0008-5472.CAN-22-1628 36318117 PMC9812290

[28] Qin Q, Popic V, Wienand K. et al. Accurate fusion transcript identification from long- and short-read isoform sequencing at bulk or single-cell resolution. Genome research 2025;35:967–86. 10.1101/gr.279200.124 40086881 PMC12047241

[29] Liu Y, Lu Z, Wang Y. et al. GFHunter enables accurate and efficient gene fusion detection in long-read cancer transcriptomes. bioRxiv 2025. 10.1101/2025.02.23.639788

[30] Heber S, Alekseyev M, Sze S-H. et al. Splicing graphs and EST assembly problem. *Bioinformatics* 2002;18:S181–8. 10.1093/bioinformatics/18.suppl_1.S181 12169546

[31] Garrison E, Sirén J, Novak AM. et al. Variation graph toolkit improves read mapping by representing genetic variation in the reference. *Nat Biotechnol* 2018;36:875–9. 10.1038/nbt.4227 30125266 PMC6126949

[32] Kim D, Paggi JM, Park C. et al. Graph-based genome alignment and genotyping with HISAT2 and HISAT-genotype. *Nat Biotechnol* 2019;37:907–15. 10.1038/s41587-019-0201-4 31375807 PMC7605509

[33] Eizenga JM, Novak AM, Sibbesen JA. et al. Pangenome graphs. *Annu Rev Genomics Hum Genet* 2020;21:139–62. 10.1146/annurev-genom-120219-080406 32453966 PMC8006571

[34] Sibbesen JA, Eizenga JM, Novak AM. et al. Haplotype-aware pantranscriptome analyses using spliced pangenome graphs. *Nat Methods* 2023;20:239–47. 10.1038/s41592-022-01731-9 36646895

[35] Hu H, Gao R, Jiang Z. et al. SVPG: a pangenome-based structural variant detection approach and rapid augmentation of pangenome graphs with new samples. bioRxiv 2025. 10.1101/2025.07.11.664486 PMC13645633 42768106

[36] Denti L, Rizzi R, Beretta S. et al. ASGAL: aligning RNA-seq data to a splicing graph to detect novel alternative splicing events. *BMC Bioinformatics* 2018;19:444–21. 10.1186/s12859-018-2436-3 30458725 PMC6247705

[37] Li H, Feng X, Chu C. The design and construction of reference pangenome graphs with minigraph. *Genome Biol* 2020;21:265–19. 10.1186/s13059-020-02168-z 33066802 PMC7568353

[38] Li HJB . Minimap2: pairwise alignment for nucleotide sequences. *Bioinformatics* 2018;34:3094–100. 10.1093/bioinformatics/bty191 29750242 PMC6137996

[39] Gao Y, Liu Y, Ma Y. et al. abPOA: an SIMD-based C library for fast partial order alignment using adaptive band. *Bioinformatics* 2021;37:2209–11. 10.1093/bioinformatics/btaa963 33165528

[40] Ono Y, Hamada M, Asai K. et al. PBSIM3: a simulator for all types of PacBio and ONT long reads. *NAR Genom Bioinformatics* 2022;4:lqac092. 10.1093/nargab/lqac092 PMC9713900 36465498

[41] Babiceanu M, Qin F, Xie Z. et al. Recurrent chimeric fusion RNAs in non-cancer tissues and cells. *Nucleic Acids Res* 2016;44:2859–72. 10.1093/nar/gkw032 26837576 PMC4824105

[42] Pintarelli G, Dassano A, Cotroneo CE. et al. Read-through transcripts in normal human lung parenchyma are down-regulated in lung adenocarcinoma. *Oncotarget* 2016;7:27889–98. 10.18632/oncotarget.8556 27058892 PMC5053695

[43] Horwitz K, Costlow M, McGuire W. MCF-7: a human breast cancer cell line with estrogen, androgen, progesterone, and glucocorticoid receptors. *Steroids* 1975;26:785–95. 10.1016/0039-128X(75)90110-5 175527

[44] Edgren H, Murumagi A, Kangaspeska S. et al. Identification of fusion genes in breast cancer by paired-end RNA-sequencing. *Genome Biol* 2011;12:R6–13. 10.1186/gb-2011-12-1-r6 21247443 PMC3091304

[45] Kangaspeska S, Hultsch S, Edgren H. et al. Reanalysis of RNA-sequencing data reveals several additional fusion genes with multiple isoforms. *PloS One* 2012;7:e48745. 10.1371/journal.pone.0048745 23119097 PMC3485361

[46] Sakarya O, Breu H, Radovich M. et al. RNA-seq mapping and detection of gene fusions with a suffix array algorithm. *PLoS Comput Biol* 2012;8:e1002464. 10.1371/journal.pcbi.1002464 22496636 PMC3320572

[47] Inaki K, Hillmer AM, Ukil L. et al. Transcriptional consequences of genomic structural aberrations in breast cancer. *Genome Res* 2011;21:676–87. 10.1101/gr.113225.110 21467264 PMC3083084

[48] Maher CA, Palanisamy N, Brenner JC. et al. Chimeric transcript discovery by paired-end transcriptome sequencing. *Proc Natl Acad Sci U S A* 2009;106:12353–8. 10.1073/pnas.0904720106 19592507 PMC2708976

[49] Asmann YW, Hossain A, Necela BM. et al. A novel bioinformatics pipeline for identification and characterization of fusion transcripts in breast cancer and normal cell lines. *Nucleic Acids Res* 2011;39:e100–0. 10.1093/nar/gkr362 21622959 PMC3159479

[50] Chen Y, Davidson NM, Wan YK. et al. A systematic benchmark of nanopore long-read RNA sequencing for transcript-level analysis in human cell lines. *Nat Methods* 2025;**22**:801-812. 10.1038/s41592-025-02623-4 PMC11978509 40082608

[51] Jang YE, Jang I, Kim S. et al. ChimerDB 40: an updated and expanded database of fusion genes. *Nucleic Acids Res* 2020;48:D817–24. 10.1093/nar/gkz1013 31680157 PMC7145594

