# Supplementary material for: GFSeeker: a splicing-graph-based approach for accurate gene fusion detection from long-read RNA sequencing data
Source: Brief Bioinform. 2026 Jan 7;27(1):bbaf702. doi: 10.1093/bib/bbaf702 (PMC12777712; doi:10.1093/bib/bbaf702)
Supplement: Supplementary_materials_bbaf702 [file supplementary_materials_bbaf702.pdf]

GFSeeker: A Splicing-Graph-Based Approach for Accurate Gene Fusion Detection from Long-Read RNA Sequencing Data

Supplementary Materials

Table S1 Statistics of simulated datasets

Coverage	Platform	Error rate	Reads	Throughput	N50
10X	ONT	5%	3,925,104	5,520,095,857	2,124
		10%	3,883,945	5,520,672,309	2,142
		15%	3,868,841	5,521,633,968	2,153
	PacBio	5%	3,936,697	5,520,404,641	2,122
		10%	3,896,405	5,520,527,881	2,137
		15%	3,867,793	5,521,866,088	2,153
30X	ONT	5%	5,773,628	8,363,022,901	2,201
		10%	5,726,966	8,363,232,386	2,224
		15%	5,706,659	8,363,751,613	2,235
	PacBio	5%	5,783,121	8,363,236,919	2,200
		10%	5,740,685	8,363,346,947	2,217
		15%	5,702,738	8,364,417,634	2,236
50X	ONT	5%	4,783,218	6,928,520,359	2,189
		10%	4,732,518	6,928,867,431	2,211
		15%	4,701,315	6,928,692,000	2,223
	PacBio	5%	4,791,206	6,928,742,383	2187
		10%	4,754,396	6,929,097,806	2,205
		15%	4,699,203	6,929,596,062	2,224

Table S2 Benchmark results on simulated datasets

Dataset	Tool	10X				30X				50X			
		TP	P	R	F1	TP	P	R	F1	TP	P	R	F1
PacBio 15% err	JAFFAL	102	0.836	0.699	0.761	111	0.835	0.760	0.796	117	0.886	0.801	0.842
	GFHunter	114	0.832	0.781	0.806	124	0.800	0.849	0.824	126	0.834	0.863	0.849
	LongGF	93	0.877	0.637	0.738	109	0.940	0.747	0.832	113	0.958	0.774	0.856
	CTAT-LR-Fusion	110	0.683	0.753	0.717	121	0.756	0.829	0.791	120	0.833	0.822	0.828
	FusionSeeker	92	0.310	0.630	0.415	108	0.417	0.740	0.533	110	0.561	0.753	0.643
	**GFSeeker**	**116**	**1.000**	**0.795**	**0.886**	**132**	**0.985**	**0.904**	**0.943**	**130**	**0.949**	**0.890**	**0.919**
PacBio 10% err	JAFFAL	115	0.816	0.788	0.801	125	0.839	0.856	0.848	126	0.881	0.863	0.872
	GFHunter	128	0.837	0.877	0.856	133	0.816	0.911	0.861	134	0.859	0.918	0.887
	LongGF	116	0.886	0.795	0.838	119	0.930	0.815	0.869	121	0.938	0.829	0.880
	CTAT-LR-Fusion	124	0.697	0.849	0.765	130	0.774	0.890	0.828	129	0.843	0.884	0.863
	FusionSeeker	111	0.356	0.760	0.485	118	0.466	0.808	0.592	119	0.598	0.815	0.690
	**GFSeeker**	**136**	**0.986**	**0.932**	**0.958**	**143**	**0.973**	**0.980**	**0.976**	**143**	**0.973**	**0.980**	**0.976**
PacBio 5% err	JAFFAL	128	0.837	0.877	0.856	129	0.854	0.884	0.869	130	0.890	0.890	0.890
	GFHunter	135	0.833	0.925	0.877	135	0.854	0.925	0.888	137	0.851	0.938	0.893
	LongGF	121	0.890	0.829	0.858	126	0.933	0.863	0.897	126	0.955	0.863	0.907
	CTAT-LR-Fusion	132	0.729	0.904	0.807	133	0.806	0.911	0.855	135	0.865	0.925	0.894
	FusionSeeker	118	0.393	0.808	0.529	122	0.511	0.836	0.634	123	0.647	0.843	0.732
	**GFSeeker**	**143**	**0.986**	**0.980**	**0.983**	**145**	**0.967**	**0.993**	**0.980**	**145**	**0.954**	**0.993**	**0.973**
ONT 15% err	JAFFAL	106	0.841	0.726	0.779	116	0.853	0.795	0.823	123	0.891	0.843	0.866
	GFHunter	123	0.843	0.843	0.843	124	0.821	0.849	0.835	127	0.825	0.870	0.847
	LongGF	107	0.892	0.733	0.805	110	0.924	0.753	0.830	118	0.944	0.808	0.871
	CTAT-LR-Fusion	118	0.756	0.808	0.782	118	0.776	0.808	0.792	127	0.836	0.870	0.852
	FusionSeeker	105	0.332	0.719	0.455	107	0.437	0.733	0.547	117	0.585	0.801	0.676
	**GFSeeker**	**123**	**0.992**	**0.843**	**0.911**	**125**	**1.000**	**0.856**	**0.923**	**140**	**0.952**	**0.959**	**0.956**
ONT 10% err	JAFFAL	127	0.830	0.870	0.850	126	0.840	0.863	0.851	131	0.885	0.897	0.891
	GFHunter	135	0.828	0.925	0.874	135	0.828	0.925	0.874	137	0.825	0.938	0.878

	LongGF	123	0.891	0.843	0.866	123	0.932	0.843	0.885	126	0.947	0.863	0.903
	CTAT-LR-Fusion	132	0.742	0.904	0.815	133	0.796	0.911	0.850	135	0.860	0.925	0.891
	FusionSeeker	120	0.387	0.822	0.526	122	0.494	0.836	0.621	123	0.609	0.843	0.707
	**GFSeeker**	**139**	**0.993**	**0.952**	**0.972**	**143**	**0.966**	**0.980**	**0.973**	**145**	**0.948**	**0.993**	**0.970**
ONT 5% err	JAFFAL	122	0.824	0.836	0.830	129	0.843	0.884	0.863	130	0.884	0.890	0.887
	GFHunter	133	0.837	0.911	0.872	136	0.861	0.932	0.895	135	0.854	0.925	0.888
	LongGF	126	0.894	0.863	0.878	125	0.933	0.856	0.893	126	0.947	0.863	0.903
	CTAT-LR-Fusion	134	0.753	0.918	0.827	135	0.839	0.925	0.880	135	0.871	0.925	0.897
	FusionSeeker	123	0.416	0.843	0.557	123	0.513	0.843	0.637	123	0.631	0.843	0.721
	**GFSeeker**	**145**	**0.986**	**0.993**	**0.990**	**145**	**0.960**	**0.993**	**0.976**	**146**	**0.954**	**1.000**	**0.977**

Table S3 Benchmark results on breakpoint evaluation of simulated datasets

Dataset	Tool	Strict				Fuzzy			
		TP	P	R	F1	TP	P	R	F1
PacBio	JAFFAL	123	0.815	0.843	0.828	127	0.841	0.870	0.855
	GFHunter	61	0.386	0.418	0.401	130	0.823	0.890	0.855
	LongGF	31	0.230	0.212	0.221	124	0.919	0.849	0.883
	CTAT-LR-Fusion	129	0.782	0.884	0.830	132	0.800	0.904	0.849
	FusionSeeker	46	0.193	0.315	0.239	119	0.498	0.815	0.618
	**GFSeeker**	**134**	**0.893**	**0.918**	**0.905**	**138**	**0.920**	**0.945**	**0.932**
ONT	JAFFAL	123	0.804	0.843	0.823	126	0.824	0.863	0.843
	GFHunter	61	0.386	0.418	0.401	131	0.829	0.897	0.862
	LongGF	33	0.246	0.226	0.236	123	0.918	0.843	0.879
	CTAT-LR-Fusion	131	0.814	0.897	0.853	134	0.832	0.918	0.873
	FusionSeeker	47	0.196	0.322	0.244	120	0.500	0.822	0.622
	**GFSeeker**	**138**	**0.914**	**0.945**	**0.929**	**142**	**0.940**	**0.973**	**0.956**

Table S4 Benchmark results on SeraCare real cancer datasets

Dataset	Tool	TP	P	R	F1
SRR27957791	JAFFAL	14	0.519	0.875	0.651
	GFHunter	14	0.778	0.875	0.824
	LongGF	13	0.591	0.813	0.684
	CTAT-LR-Fusion	15	0.682	0.938	0.790
	FusionSeeker	14	0.139	0.875	0.239
	**GFSeeker**	**16**	**0.842**	**1**	**0.914**
SRR27957792	JAFFAL	14	0.452	0.875	0.596
	GFHunter	14	0.609	0.875	0.718
	LongGF	13	0.464	0.813	0.591
	CTAT-LR-Fusion	15	0.600	0.938	0.732
	FusionSeeker	14	0.105	0.875	0.188
	**GFSeeker**	**16**	**0.800**	**1**	**0.889**
SRR27957793	JAFFAL	14	0.412	0.875	0.560
	GFHunter	14	0.667	0.875	0.757
	LongGF	13	0.351	0.813	0.491
	CTAT-LR-Fusion	15	0.556	0.938	0.698
	FusionSeeker	14	0.087	0.875	0.158
	**GFSeeker**	**16**	**0.762**	**1**	**0.865**

Table S5 Benchmark results on breakpoint evaluation of SeraCare real cancer datasets

Dataset	Tool	Strict				Fuzzy			
		TP	P	R	F1	TP	P	R	F1
SRR27957791	JAFFAL	13	0.482	0.813	0.605	14	0.519	0.875	0.651
	GFHunter	8	0.444	0.500	0.471	13	0.722	0.813	0.765
	LongGF	2	0.091	0.125	0.105	12	0.546	0.750	0.632
	CTAT-LR-Fusion	15	0.682	0.938	0.790	15	0.682	0.938	0.790
	FusionSeeker	5	0.050	0.313	0.086	14	0.139	0.875	0.239
	**GFSeeker**	**15**	**0.790**	**0.938**	**0.857**	**15**	**0.790**	**0.938**	**0.857**
SRR27957792	JAFFAL	13	0.419	0.813	0.553	14	0.452	0.875	0.596
	GFHunter	8	0.348	0.500	0.410	13	0.565	0.813	0.667
	LongGF	5	0.179	0.313	0.227	12	0.429	0.750	0.546
	CTAT-LR-Fusion	15	0.600	0.938	0.732	15	0.600	0.938	0.732
	FusionSeeker	5	0.038	0.313	0.067	14	0.105	0.875	0.188
	**GFSeeker**	**15**	**0.750**	**0.938**	**0.833**	**15**	**0.750**	**0.938**	**0.833**
SRR27957793	JAFFAL	13	0.382	0.813	0.520	14	0.412	0.875	0.560
	GFHunter	8	0.381	0.500	0.432	13	0.619	0.813	0.703
	LongGF	4	0.108	0.250	0.151	12	0.324	0.750	0.453
	CTAT-LR-Fusion	15	0.556	0.938	0.698	15	0.556	0.938	0.698
	FusionSeeker	5	0.031	0.313	0.057	14	0.087	0.875	0.158
	**GFSeeker**	**15**	**0.714**	**0.938**	**0.811**	**15**	**0.714**	**0.938**	**0.811**

Table S6 Benchmark results on MCF-7 real cancer datasets

Dataset	Tool	TP	P	R	F1
MCF7_cDNA	JAFFAL	9	0.450	0.220	0.295
	GFHunter	1	1	0.024	0.048
	LongGF	10	0.417	0.244	0.308
	CTAT-LR-Fusion	11	0.423	0.268	0.328
	FusionSeeker	9	0.114	0.220	0.150
	**GFSeeker**	**12**	**0.706**	**0.293**	**0.414**
MCF7_directcDNA	JAFFAL	19	0.463	0.463	0.463
	GFHunter	3	1	0.073	0.136
	LongGF	19	0.153	0.463	0.230
	CTAT-LR-Fusion	17	0.085	0.415	0.141
	FusionSeeker	18	0.008	0.439	0.015
	**GFSeeker**	**20**	**0.645**	**0.488**	**0.556**
MCF7_RNA_1	JAFFAL	12	0.522	0.293	0.375
	GFHunter	3	0.750	0.073	0.133
	LongGF	13	0.542	0.317	0.400
	CTAT-LR-Fusion	14	0.500	0.342	0.406
	FusionSeeker	15	0.217	0.366	0.273
	**GFSeeker**	**16**	**0.640**	**0.390**	**0.485**
MCF7_RNA_2	JAFFAL	12	0.632	0.293	0.400
	GFHunter	0	0	0	0
	LongGF	12	0.632	0.293	0.400
	CTAT-LR-Fusion	13	0.684	0.317	0.433
	FusionSeeker	11	0.186	0.268	0.220
	**GFSeeker**	**13**	**0.684**	**0.317**	**0.433**

Table S7 Ground truth list of MCF-7 real cancer datasets

	Gene1*	Gene2*
1	*BCAS4*	*BCAS3*
2	*ARFGEF2*	*SULF2*
3	*RPS6KB1*	*VMP1*
4	*SMARCA4*	*CARM1*
5	*SLC25A24*	*NBPF6*
6	*USP31*	*CRYL1*
7	*TBL1XR1*	*RGS17*
8	*TAF4*	*BRIP1*
9	*RPS6KB1*	*DIAPH3*
10	*AHCYL1*	*RAD51C*
11	*TXLNG*	*SYAP1*
12	*MYO6*	*SENP6*
13	*POP1*	*MATN2*
14	*GATAD2B*	*NUP210L*
15	*ESR1*	*CCDC170*
16	*DEPDC1B*	*ELOVL7*
17	*ATXN7L3*	*FAM171A2*
18	*SYTL2*	*PICALM*
19	*ADAMTS19*	*SLC27A6*
20	*ARHGAP19*	*DRG1*
21	*MYO9B*	*FCHO1*
22	*PAPOLA*	*AK7*
23	*ATP1A1*	*ZFP64*
24	*B3GNTL1*	*SLC9A8*
25	*BCAS3*	*AMPD1*
26	*BCAS3*	*ATXN7*
27	*BCAS4*	*ZMYND8*
28	*CHEK2*	*XBP1*
29	*KCND3*	*PPM1E*
30	*NAV1*	*GPR37L1*
31	*NCOA3*	*SULF2*
32	*PLCG1*	*TOP1*
33	*PNPLA7*	*DPH7*
34	*RAD51C*	*ATXN7*
35	*SGPP2*	*ULK4*
36	*SULF2*	*PRICKLE2*
37	*TEX14*	*PTPRG*
38	*TSPAN9*	*TEAD4*
39	*UBE2V1*	*TBX2*
40	*ZMYND8*	*USP32*
41	*MYH9*	*EIF3D*

*Source of Ground truth list is from reference [1-6].

Table S8 Statistics of real cancer datasets and non-tumor datasets

Dataset	Reads	Throughput	Average error rate	N50
SGNex_MCF7_cDNA_replicate1_run3	2,849,147	2,154,260,463	9.61%	3,932
SGNex_MCF7_directcDNA_replicate4_run2	4,948,000	6,810,167,086	11.86%	1,709
SGNex_MCF7_directRNA_replicate2_run3	7,230,921	5,272,174,709	15.15%	6,134
SGNex_MCF7_directRNA_replicate4_run1	1,380,111	1,380,687,881	9.89%	1,359
SRR27957791	7,542,912	8,089,585,160	0.37%	1,299
SRR27957792	7,825,468	8,319,067,878	0.38%	1,280
SRR27957793	4,694,529	6,171,220,054	0.44%	1,561
NA24385_cDNA	57,355,400	34,328,999,706	4.18%	832
NA24631_dRNA	2,805,063	2,581,350,920	9.55%	1,096
m64139_220127_180020_hifi_reads	4,434,560	9,103,769,516	0.68%	2,539

Table S9 Data availability

No.	Dataset	Type	Accession
1	GRCh38.p14.genome.fa	Reference genome	https://ftp.ebi.ac.uk/pub/databases/gencode/Gencode_human/release_47/GRCh38.p14.genome.fa.gz
2	gencode.v47.annotation.gff3	Gene annotation	https://ftp.ebi.ac.uk/pub/databases/gencode/Gencode_human/release_47/gencode.v47.annotation.gff3.gz
3	HG002 Iso-seq	Real non-tumor dataset	https://ftp-trace.ncbi.nlm.nih.gov/ReferenceSamples/giab/data_RNAseq/AshkenazimTrio/HG002_NA24385_son/Baylor_PacBio/reads/m64139_220127_180020.hifi_reads.bam
4	HG002 cDNA	Real non-tumor dataset	https://s3.amazonaws.com/gtl-public-data/giab/ONT_dRNA/03_30_23_R941_DRS_NA24385_dRNA_Guppy_6.4.6_rna_hac_prom.pass.NoU.fastq.gz
5	HG002 dRNA	Real non-tumor dataset	https://s3.amazonaws.com/gtl-public-data/giab/ONT_dRNA/03_30_23_R941_DRS_NA24631_dRNA_Guppy_6.4.6_rna_hac_prom.pass.NoU.fastq.gz
6	MCF-7 cDNA	Real tumor dataset	s3://sg-nex-data/data/sequencing_data_ont/fastq/SGNex_MCF7_cDNA_replicate1_run3/SGNex_MCF7_cDNA_replicate1_run3.fastq.gz
7	MCF-7 directcDNA	Real tumor dataset	s3://sg-nex-data/data/sequencing_data_ont/fastq/SGNex_MCF7_directcDNA_replicate4_run2/SGNex_MCF7_directcDNA_replicate4_run2.fastq.gz
8	MCF-7 dRNA	Real tumor dataset	s3://sg-nex-data/data/sequencing_data_ont/fastq/SGNex_MCF7_directRNA_replicate2_run3/SGNex_MCF7_directRNA_replicate2_run3.fastq.gz<br>s3://sg-nex-data/data/sequencing_data_ont/fastq/SGNex_MCF7_directRNA_replicate4_run1/SGNex_MCF7_directRNA_replicate4_run1.fastq.gz
9	SeraCare Iso-seq	Real fusion dataset	SRR27957791<br>SRR27957792<br>SRR27957793

Table S10 All the tools used in this study

Tools	Version	URL	Category
JAFFAL	v2.3	https://github.com/Oshlack/JAFFA	Long read fusion detection tools
GFHunter	v1.0.0	https://github.com/luzhenhao-HIT/GFHunter	Long read fusion detection tools
LongGF	v0.1.2	https://github.com/WGLab/LongGF	Long read fusion detection tools
CTAT-LR-Fusion	v1.1.0	https://github.com/TrinityCTAT/CTAT-LR-fusion	Long read fusion detection tools
FusionSeeker	v1.0.1	https://github.com/Maggi-Chen/FusionSeeker	Long read fusion detection tools
Minimap2	v2.28-r1209	https://github.com/lh3/minimap2	Long read sequence aligner
mappy	v2.29	https://github.com/lh3/minimap2	Long read sequence aligner
minigraph	v0.21-r606	https://github.com/lh3/minigraph	Sequence-to-graph mapper
abPOA	v1.5.3	https://github.com/yangao07/abPOA	Multiple sequence aligner
PBSIM3	v3.0.5	https://github.com/yukiteruono/pbsim3	Long read sequencing simulator
Samtools	v1.2.1	https://github.com/samtools/samtools	SAM processing tool

Supplementary Notes

1. Metrics for the evaluation

To quantify and evaluate the performance of different gene fusion detection tools on different datasets, we evaluated the precision, recall, and F1 of each fusion detection method by comparing their predictions with the respectively defined true value sets in the following way, which are defined as follows:

$$precision = \frac{TP}{TP + FP} \tag{1}$$

$$recall = \frac{TP}{TP + FN} \tag{2}$$

$$F1 = 2 \times \frac{precision \times recall}{precision + recall} \tag{3}$$

where TP (true positive) and FP (false positive) represent the number of fusions that match/mismatch the ground truth. FN (false negative) represent the number of fusions in the ground truth that are unreported.

2. Detailed description of the five criteria for targeted ligation validation and filtering:

1) Structural Consistency Constraints: The positions of the alignment segments on the two gene subgraphs must be plausible and form a continuous transcript structure within the original read, with no excessive overlap or gap between them.

2) Transcriptional Direction Consistency: To ensure biological plausibility, the alignment to both genes must be consistent with their native transcriptional direction (i.e., on the sense strand). GFSeeker verifies this by comparing the read's path direction to each gene's known genomic strand, which effectively filters out biologically meaningless connections (e.g., one gene forward, one reverse) and is essential for preserving a continuous Open Reading Frame (ORF) in the potential chimeric transcript.

3) Minimum Block Length: Each continuous aligned segment (block) on a gene must meet a minimum length threshold (default: 25 bp) to exclude noise from short, potentially random sequence matches.

4)Validation of the Fusion Junction: The validity of the fusion junction is confirmed by analyzing both the initial graph alignment path (from the GAF file) and the targeted mapping results. This ensures the read accurately connects the boundaries of the two genes in a manner consistent with a splicing pattern.

5)Removal of Alignment Anomalies: The mapping must be free of significant anomalies, which excludes reads that fail to align correctly to the custom reference or whose mapping results differ significantly from the original graph alignment.

3. Three different types of MCF-7 Cancer Cell Line ONT datasets:

1) Direct RNA sequencing (dRNA): Comprising two biological replicates, this technique sequences RNA molecules directly, preserving original modification information and avoiding PCR amplification bias.

2) Direct cDNA sequencing (d-cDNA): Comprising one biological sample, this technique sequences full-length cDNA, also avoiding PCR amplification.

3) Standard cDNA sequencing (std-cDNA): Comprising one biological sample, this technique involves PCR amplification after reverse transcription, a common source of chimeric artifacts.

4. The "min-support" parameter of supporting reads note:

For all the simulated datasets, minimum supported readings for all tools were set to 2. For the HG002 none-tumor sample's PacBio Iso-Seq/ONT dRNA/ONT cDNA data, minimum supported readings for all tools were set to 3/3/6. For the MAS-ISO-seq Seraseq Fusion RNA Mix v4 data, minimum supported readings for all tools were set to 3. For the MCF-7 ONT dRNA/ONT cDNA data, minimum supported readings for all tools were set to 3.

5. Construction of simulation data:

5.1 Positive simulations on ONT

```
pbsim --strategy templ \
      --method errhmm \
      --errhmm ERRHMM-ONT.model \
      --accuracy-mean 0.85 \
      --prefix GF \
      --id-prefix GF \
      --pass-num depth \
      --template sim_template.fasta
pbsim --strategy templ \
      --method errhmm \
      --errhmm ERRHMM-ONT-HQ.model \
      --accuracy-mean 0.90 or 0.95 \
      --prefix GF \
      --id-prefix GF \
      --pass-num depth \
      --template sim_template.fasta
samtools fasta GF.sam > ont_simulation_depth.fasta
```

5.2 Positive simulations on PacBio

```
pbsim --strategy templ \
      --method errhmm \
      --errhmm ERRHMM- RSII.model \
      --accuracy-mean 0.85 \
```

```
        --prefix GF \
        --id-prefix GF \
        --pass-num depth \
        --template sim_template.fasta
pbsim --strategy templ \
        --method errhmm \
        --errhmm ERRHMM- SEQUEL.model \
        --accuracy-mean 0.90 or 0.95 \
        --prefix GF \
        --id-prefix GF \
        --pass-num depth \
        --template sim_template.fasta
samtools fasta GF.sam > pacbio_simulation_depth.fasta
```

5.3 Negative simulations on ONT

```
type="Normal"
threads=n
run_simulation() {
    i=$1
    num=$(printf "%03d" "$i")
    name="${type}${num}"
    pbsim --strategy wgs \
        --method qshmm \
        --qshmm QSHMM-ONT.model \
        --depth depth \
        --prefix "$name" \
        --accuracy-mean 0.85 \
        --id-prefix "Normal${num}_" \
        --genome " gencode.v47.len_over100_filtered_transcripts.part_${num}.fa"
    rm -f "${name}"*.maf "${name}"*.ref
    cat "${name}"*.fastq > "all_${i}.fastq"
    rm -f "${name}"*.fastq
```

```
    samtools fasta "all_${i}.fastq" > "all_${i}.fasta"
     rm "all_${i}.fastq"
}
export -f run_simulation
export type
seq 1 40 | parallel -j $threads run_simulation
cat all_*.fasta > ../Normal_ONT_depth.fasta
rm -f all_*.fasta
```

5.4 Negative simulations on PacBio

```
type="Normal"
threads=n
run_simulation() {
    i=$1
    num=$(printf "%03d" "$i")
    name="${type}${num}"
    pbsim --strategy wgs \
          --method qshmm \
          --qshmm QSHMM-RSII.model \
          --depth depth \
          --prefix "$name" \
          --accuracy-mean 0.85 \
          --id-prefix "Normal${num}_" \
          --genome " gencode.v47.len_over100_filtered_transcripts.part_${num}.fa"
    rm -f "${name}"*.maf "${name}"*.ref
    cat "${name}"*.fastq > "all_${i}.fastq"
    rm -f "${name}"*.fastq
    samtools fasta "all_${i}.fastq" > "all_${i}.fasta"
     rm "all_${i}.fastq"
}
export -f run_simulation
export type
```

```
seq 1 40 | parallel -j $threads run_simulation
cat all_*.fasta > ../Normal_PacBio_depth.fasta
rm -f all_*.fasta
```

5.5 Merge positive simulations and negative simulations

```
cat Normal_PacBio_depth.fasta pacbio_simulation_depth.fasta > simfusion_PacBio_depth.fasta
cat Normal_ONT_depth.fasta ont_simulation_depth.fasta > simfusion_ONT_depth.fasta
```

6. The command lines for fusion identification

GFSeeker

```
python GFSeeker graph \
    gencode.v47.annotation.gff3 \
    gencode.GRCh38.p14.genome.fa
python GFSeeker detect \
    XXX.fasta \
    gencode.GRCh38.p14.genome.fa \
    ./graph_ref/
```

JAFFAL

```
docker run \
    -v :/ref -v :/results -v :/data \
    -w davidsongroup/jaffa:latest \
    bpipe run -n 32 \
    /JAFFA/JAFFAL.groovy /data/XXX.fasta
```

GFHunter

```
GFHunter index \
    gencode.v47.annotation.gtf \
    gencode.GRCh38.p14.genome.fa \
    ./index/
GFHunter detect \
    -o name \
    -t 32 \
    -M \
    XXX.fasta \
```

./index/

LongGF

```
minimap2 -ax splice \
    -t 32 \
    ref_index.mmi \
    XXX.fasta \
    -o .sam
samtools view -b .sam -o .bam
LongGF .bam \
    gencode.v47.annotation.gtf \
    100 30 100 \
    > name.log
```

CTAT-LR-Fusion

```
docker run \
    -v :/datasource -v :/output -v:/ctat_genome_lib \
    trinityctat/ctat_lr_fusion:latest \
    ctat-LR-fusion -T /datasource/XXX.fasta \
    --genome_lib_dir/ctat_genome_lib \
    -o /output/XXX \
    --CPU 32
```

FusionSeeker

```
minimap2 -ax splice \
    -t 32 \
    ref_index.mmi \
    XXX.fasta \
    -o .sam
samtools sort .sam -o .bam
samtools index .bam
FusionSeeker --bam .bam \
    -o FusionSeeker_out/ \
    --gtf gencode.v47.annotation.gtf \
```

```
--ref gencode.GRCh38.p14.genome.fa \
--thread 32
```

Reference

1. Edgren H, Murumagi A, Kangaspeska S et al. Identification of fusion genes in breast cancer by paired-end RNA-sequencing 2011;12:1-13.

2. Kangaspeska S, Hultsch S, Edgren H et al. Reanalysis of RNA-sequencing data reveals several additional fusion genes with multiple isoforms 2012;7:e48745.

3. Sakarya O, Breu H, Radovich M et al. RNA-Seq mapping and detection of gene fusions with a suffix array algorithm 2012;8:e1002464.

4. Inaki K, Hillmer AM, Ukil L et al. Transcriptional consequences of genomic structural aberrations in breast cancer 2011;21:676-687.

5. Maher CA, Palanisamy N, Brenner JC et al. Chimeric transcript discovery by paired-end transcriptome sequencing 2009;106:12353-12358.

6. Asmann YW, Hossain A, Necela BM et al. A novel bioinformatics pipeline for identification and characterization of fusion transcripts in breast cancer and normal cell lines 2011;39:e100-e100.
